# On the Construction and Management of Hospitals for the Insane; with a Particular Notice of the Institution of Siegburg

**Published:** 1842-01

**Authors:** 


ART. I.—1. *On the Construction and Management of Hospitals for the Insane; with a particular notice of the Institution of Siegburg.* By Dr. MAXIMILIAN JACOBI. Translated (from the German) by JOHN KITCHING. With Introductory Observations by SAMUEL TUKE.—*London,* 1841. 8vo.

2. *The Statistics of the Retreat; consisting of a Report and Tables exhibiting the Experience of that Institution for the Insane, from its establishment in* 1796 *to* 1840.—*York,* 1841. 8vo, pp. 62, with 51 Tables.

3. *The Fifty-ninth Report of the Visiting Justices for the County Lunatic Asylum at Hanwell; and Third Report of the Resident Physician,* JOHN CONOLLY, M.D.—*London,* 1841. 8vo, pp. 137.

THESE three works are among the most valuable documents on the subject of insanity that have made their appearance for many years; and we hope, on a future occasion, to enter upon a full consideration of the most important subjects treated in them. At present we can scarcely do more than direct the attention of our readers to them.

1. Jacobi's original work is a useful production; the present translation of it is faithfully executed; and enriched as it now is by notes and a long introduction from the pen of Mr. Tuke, it cannot fail to be appreciated as it deserves. It ought, indeed, to be in the hands of every one connected with the projecting, planning, or managing of asylums. In the introduction we have much valuable original matter on the external and internal arrangements for the government of establishments for the insane, on the construction of asylums, and on the statistics of insanity. All these subjects are treated with that sound judgment, cool impartiality, good sense, and benevolent feeling, which characterize the productions of Mr. Tuke, to whom science and humanity must ever remain deeply indebted for being the first in this country to open the way to that mild and just treatment of the insane, which has been so gloriously consummated by Dr. Conolly. The two following remarks in Mr. Tuke's essay are exactly applicable to the authors of this book in its present form, and to the book itself; and commending them to the attention of our readers we shall, for the present, close our notice of the work.

"They who have taken right theoretical views of disordered mental affections may be prepared to state generally the provisions which should be made for their treatment in public or private establishments; but it is only those who have practically observed the circumstances of the insane, in establishments provided for them, and who have corrected their theoretical inferences by the test of experience and comparative observation, who can supply that particular information which we require in the consideration of the best means of providing for the wants of the insane; whether as regards the construction of their abodes, or those various arrangements and administrations which constitute the economy of hospitals devoted to this class of sufferers.

"If those who are engaged in the chief management of the insane, in our public establishments, would use this work as a text-book for their enquiries and considerations, carefully noting the results of their observations, we might, before long, without aiming at an exact prescription in these matters, arrive at some sound conclusions, in regard to the construction of the buildings; as well as to the arrangements, external and internal, which may best provide for the right government and general economy of these establishments." (pp. 1-2.)

2. "The Statistics of the Retreat" is an elaborate and valuable production, and reflects the greatest credit on its author, Mr. Thurnam, the resident surgeon of that institution. The tables, fifty-one in number, are very comprehensive, and, together with the report which accompanies them, afford considerable information of the most authentic kind. They exhibit the results obtained at the asylum during the forty-four years that it has been in operation. The only thing to be regretted in relation to the statistics is the small number of instances on which the results are based. The average number of cases in the asylum at one time was only 67, and the total number of admissions during the 44 years only 615; the total number of persons admitted was, indeed, only 469, 146 of the cases being readmissions. The long period of time over which the experience of the Retreat extends, the uncommon precision of the knowledge obtained respecting the individual cases, and the extreme accuracy and caution with which the conclusions have been drawn, in some measure compensate for the limitation of the data; and give the whole document a standard value of no ordinary kind.

3. The degree of estimation in which we hold the Hanwell Report is best declared by the fact that we have extracted, in the Fourth Part of the present Number of our Journal, a large portion of Dr. Conolly's report, and a part of that of the magistrates. To these documents we refer our readers for proofs of the vast improvements introduced into the modes of treating the insane in this country in recent times, and especially for irrefragable evidence of the complete success of the system of universal non-restraint introduced into Hanwell by Dr. Conolly. This system has been now two complete years in operation; and every succeeding month has furnished fresh illustrations of its superiority. And when the great extent of the establishment at Hanwell is considered, and the perfect publicity of all the doings within its walls, it is impossible not to accord to the results of the experience afforded by it a degree of importance not easily over-rated. It appears from the magistrates' report that the total number of patients in the asylum on 30th September, 1840, was 858, and on 30th September, 1841, 918; the daily average number throughout the year being 883, and during the last quarter 915. And of this number not an individual has been in personal restraint during the last two years!

